# Effects of Lactic Acid Bacteria Additives on the Quality, Volatile Chemicals and Microbial Community of *Leymus chinensis* Silage During Aerobic Exposure

**DOI:** 10.3389/fmicb.2022.938153

**Published:** 2022-09-02

**Authors:** Yichao Liu, Yuyu Li, Qiang Lu, Lin Sun, Shuai Du, Tingyu Liu, Meiling Hou, Gentu Ge, Zhijun Wang, Yushan Jia

**Affiliations:** ^1^Key Laboratory of Forage Cultivation, Processing and High Efficient Utilization of Ministry of Agriculture and Rural Affairs, Inner Mongolia Agricultural University, Hohhot, China; ^2^Key Laboratory of Grassland Resources of Ministry of Education, Inner Mongolia Agricultural University, Hohhot, China; ^3^Inner Mongolia Academy of Agricultural and Animal Husbandry Sciences, Hohhot, China; ^4^National Engineering Laboratory of Biological Feed Safety and Pollution Prevention and Control, Key Laboratory of Animal Nutrition and Feed Science of Zhejiang Province, Institute of Feed Science, Zhejiang University, Hangzhou, China; ^5^College of Agriculture, Inner Mongolia University for Nationalities, Tongliao, China

**Keywords:** lactic acid bacteria, aerobic stability, microbial community, volatile chemicals, silage

## Abstract

Silage exposed to air is prone to deterioration and production of unpleasant volatile chemicals that can seriously affect livestock intake and health. The aim of this study was to investigate the effects of *Lactobacillus plantarum* (LP), *Lactobacillus buchneri* (LB), and a combination of LP and LB (PB) on the quality, microbial community and volatile chemicals of *Leymus chinensis* silage at 0, 4, and 8 days after aerobic exposure. During aerobic exposure, LP had higher WSC and LA contents but had the least aerobic stability, with more harmful microorganisms such as *Penicillium* and *Monascus* and produced more volatile chemicals such as Isospathulenol and 2-Furancarbinol. LB slowed down the rise in pH, produced more acetic acid and effectively improved aerobic stability, while the effect of these two additives combined was intermediate between that of each additive alone. Correlation analysis showed that *Actinomyces*, *Sphingomonas*, *Penicillium*, and *Monascus* were associated with aerobic deterioration, and *Weissella*, *Pediococcus*, *Botryosphaeria*, and *Monascus* were associated with volatile chemicals. In conclusion, LB preserved the quality of *L. chinensis* silage during aerobic exposure, while LP accelerated aerobic deterioration.

## Introduction

*Leymus chinensis* is widely distributed in the meadow steppe and arid steppes in eastern Eurasia due to its strong drought and cold tolerance (Zhao et al., 2016). The annual growth cycle begins in early spring and senescence occurs in late fall, so the plant can provide green forage for a long period (Song et al., 2016). As the dominant grass in the grasslands of northern China, *L. chinensis* is an important feed resource for domestic livestock and is commonly used for hay preparation. However, the quality of hay is greatly affected by the weather, and rain during the drying process can cause significant nutrient loss (Kebede et al., 2016). This problem can be effectively alleviated through silage preparation.

Ensiling can retain nutrients to the greatest extent while improving the palatability of forage and prolonging the storage time (Dong et al., 2020). However, silage easily deteriorates during the use process, as harmful microorganisms utilize organic substances such as soluble carbohydrates and proteins as substrates for growth and metabolism, which depletes silage nutrients and reduces its use value. The process also produces toxic substances and unpleasant volatile chemicals that affect livestock feed intake and palatability (Zhang et al., 2021). Some components may also enter milk and meat through livestock metabolism, resulting in changes in flavor and even harm to livestock and human health (Kung et al., 2018), so it is necessary to control the aerobic deterioration of silage.

Aerobic deterioration is usually related to the degree of fermentation and the number of harmful microorganisms when the silage is opened (Borreani et al., 2018), so one option is to add lactic acid bacteria when making silage to improve the fermentation effect of silage and inhibit the growth of undesirable bacteria (Irawan et al., 2021). Lactic acid bacteria additives induce different types of fermentation *Lactobacillus plantarum* undergoes homolactic fermentation, while *Lactobacillus buchneri* undergoes heterogeneous fermentation (Addah et al., 2014; Li et al., 2021). The product of homolactic fermentation is lactic acid, which rapidly reduces the pH value of the silage, thereby inhibiting the activities of other microorganisms (Tao et al., 2021; Xie et al., 2021). However, in addition to lactic acid, heterogeneous fermentation also produces substances such as ethanol, acetic acid, and carbon dioxide, among which acetic acid can effectively inhibit the reproduction of harmful microorganisms (Si et al., 2018; Wang et al., 2021). *Lactobacillus plantarum* additives are commonly added in silage production and can reduce dry matter loss, and improve *in vitro* digestibility and palatability (Direkvandi et al., 2021; Wei et al., 2021). However, some studies have shown that after adding *Lactobacillus plantarum*, the aerobic stability of silage may worsen. This is possibly due to the production of large amounts of lactic acid, which provides fermentation substrates for yeasts and aerobic bacteria during aerobic exposure (Yuan et al., 2016; Mugabe et al., 2020). Other studies have shown that after using *Lactobacillus buchneri*, the aerobic stability of silage is improved, possibly due to the production of large amount of acetic acid is produced, which can inhibit the growth of molds and yeasts during aerobic exposure (Nair et al., 2020; Ferrero et al., 2021). Most of the current studies on *L. chinensis* silage have investigated the effects of additives on quality and aerobic stability. Zhang et al. (2018) showed that the addition of lactic acid bacteria and water promoted microbial succession during the early stages of fermentation. Xu et al. (2021) showed that the addition of sodium benzoate and acetic acid improved quality and aerobic stability. There are few studies on the dynamics of quality, volatile chemicals and microorganisms in *L. chinensis* silage during aerobic exposure, and the relationship between them is unclear. This study investigated the effects of *Lactobacillus plantarum* and *Lactobacillus buchneri* on *L. chinensis* silage quality, volatile chemicals, and microbial community during aerobic exposure. The analysis of the relationship between various substances and microbial communities can further provide a reference for method to improve the quality and aerobic stability of silage during aerobic exposure. Our hypothesis is that lactic acid bacteria additives affect the quality, volatile chemicals and aerobic stability of *L. chinensis* silage and that their differences are related to microbial community composition during aerobic exposure.

## Materials and Methods

### Silage Preparation

The variety of *Leymus chinensis* used was Jisheng No. 1, which was planted in the Science and Technology Park of Inner Mongolia University for Nationalities in Tongliao City, Inner Mongolia Autonomous Region (E43°59′, N122°11′). The seeds were sown in late July 2018 and the material was cut into about 2 cm with a guillotine after harvesting on June 24, 2021 at the tassel stage. Fresh samples of *L. chinensis* were harvested from three locations in the same field as replicates, and the chopped *L. chinensis* from each location was mixed thoroughly and divided into aliquots for different treatments: uninoculated control group (CK), *Lactobacillus plantarum* (LP), *Lactobacillus buchneri* (LB), a combination of *Lactobacillus plantarum* and *Lactobacillus buchneri* (PB, 1:1). 0.01 g of lactic acid bacteria additives per kg of silage raw material with an inoculum of 1 × 10^6^ cfu/g (all provided by Shandong Zhongke Jiayi Biological Engineering Co., Ltd., Qingzhou, China), and the control group had the same amount of distilled water added, and we controlled the water content of the raw material to about 60%. After mixing it well, 300 g were put into a silage bag (Size: 30 cm × 40 cm; provided by Shijiazhuang Youlang Trading Co., Ltd.-Room 0802-1, Unit 04, Zhongchu Plaza, No. 198, Zhonghua North Street, Xinhua District, Shijiazhuang City, Hebei Province) and vacuum sealed. There were three repetitions of each treatment, and all samples were stored at room temperature (20–30°C) for 60 days.

### Aerobic Stability

Silage samples were opened after 60 days and put into 1 L sterile bottles, and a multi-channel data logger (Model: MDL-1048A; Shanghai Tianhe Automation Instrument Co., Ltd., China) was inserted in the center of the bottle to measure temperature and assess aerobic stability. Two layers of gauze were placed on each sterile bottle to avoid contamination by impurities and water loss. In this study, aerobic stability was defined as the time required for the silage temperature to exceed 2°C above room temperature (Ranjit and Kung, 2000).

### Chemical Composition

For chemical analysis, 150 g samples were taken at 0, 2, 4, 6, and 8 days, and dried in an oven at 65°C for 48 h to a constant weight. The dried samples were crushed, passed through a 1 mm sieve, and stored in a sealed bag. The dry matter content was determined after drying at 65°C for 48 h (Zhang et al., 2016). Crude protein (CP) was determined by the Kjeldahl method (Sun et al., 2021), crude fat (EE) was measured using an ANKOM fat analyzer (Model: XT15i; Beijing Anke Borui Technology Co., Ltd., China), acid detergent fiber (ADF) and neutral detergent fiber (NDF) were measured using an ANKOM fiber analyzer (Model: A2000i; Beijing Anke Borui Technology Co., Ltd., China), and soluble carbohydrate (WSC) was measured using anthrone-sulfuric acid colorimetry (Murphy, 1958).

### Fermentation Composition

Ten grams samples were taken at 0, 2, 4, 6, and 8 days, and 90 mL distilled water was added, tapped on a homogenizing tapper for 2 min and filtered to obtain the liquid extract (Model: LC-11L, Shanghai Jingxin Industrial Development Co., Ltd., China). The pH value was measured with an acidity meter (Model: LEICI PHS-3C, Shanghai Yitian Scientific Instrument Co., Ltd., China), the content of lactic acid (LA), acetic acid (AA), propionic acid (PA), and butyric acid (BA) were measured by high performance liquid chromatograph (Model: Waters e2695, MA, United States), and the content of ammonia nitrogen (NH_3_-N) was measured by phenol-Hypochlorous acid colorimetric determination (Broderick and Kang, 1980).

### Microbial Counting and Sequencing

Ten grams of *L. chinensis* fresh samples were taken, 90 mL of sterile water was added, and beaten with a homogenizer for 2 min to obtain bacterial liquid. MRS medium was used for lactic acid bacteria culture, potato dextrose agar medium was used for molds and yeasts culture, nutrient agar medium was used for aerobic bacteria culture, and eosin-methylene blue agar medium was used for coliform bacteria culture. The number of microbial colonies was determined by the plate count method.

Samples at 0 (CK0, LP0, LB0, and PB0), 4 (CK4, LP4, LB4, and PB4), and 8 (CK8, LP8, LB8, and PB8) days after aerobic exposure and fresh samples (X) were analyzed for microbiota by LC-Bio Technology Co., Ltd., Hangzhou, Zhejiang Province, China. Using CTAB’s DNA extraction method, microbial DNA was analyzed using paired-end Illumina sequencing on the NovaSeq platform according to the manufacturer’s recommendations, provided by LC-Bio. Bacteria 16S rDNA amplicon sequencing was performed using 341F (5′-CCTACGGGNGGCWGCAG-3′) and 805R (5′-GACTACHVGGGTATCTAATCC-3′) universal primer and fungal ITS amplicons were sequenced using ITS1 (5′-GTGARTCATCGAATCTTTG-3′) and ITS2 (5′-TCCTCCGCTTATTGATATGC-3′) universal primer. Raw reads were quality filtered according to fqtrim (v0.94) under specific filter conditions to obtain high quality clean labels. Chimeric sequences were filtered using Vsearch software (v2.3.4). After de-replication using DADA2, we obtained the feature table and feature sequence. Alpha-diversity and beta-diversity were calculated by random normalization to the same sequence. Feature abundances were then normalized using the relative abundance of each sample according to the SILVA (release 138) classifier. Alpha diversity was applied to the complexity analysis of sample species diversity through five indicators including Chao1, OTUs, Goods coverage, Shannon, Simpson, and all these indicators in our sample were calculated with QIIME2.

### Volatile Chemicals Analysis by GC-MS

Samples were taken at 0, 4, and 8 days of aerobic exposure of *L. chinensis* silage, snap frozen in liquid nitrogen and transferred to −80°C refrigerator for storage. The samples were ground to a powder in liquid nitrogen and 2 g of the powder was immediately transferred to a 20 mL headspace vial and sealed with a gasket and aluminum cap. The headspace vial was placed on a magnetic heating stirrer to evenly heat it. The aged extraction head was inserted into the headspace vial, and the fiber head was exposed in the headspace bottle to fully absorb the volatile chemicals of *L. chinensis* silage. The extraction temperature was 125°C, and the adsorption time was 50 min. After sufficient adsorption, the extraction head was quickly inserted into the GC-MS injection port so that the carrier gas continuously blew the volatile chemicals adsorbed on the fiber head into the GC-MS for analysis. Chromatographic conditions: an HP-5MS UI quartz capillary column (30 m × 0.25 mm × 0.25 μm) was used, the initial temperature of the column was 35°C maintained for 4 min, and the temperature was programmed to 200°C at 5°C min^–1^ maintained for 5 min, after which temperature was increased to 250°C by a program of 15°C min^–1^ and held for 4 min. The temperature of the injection port was 250°C, the carrier gas was high-purity helium (purity ≥99.99%) and the flow rate of helium was 1 mL min^–1^, the precolumn pressure was 87.57 kPa, the injection mode was split with a split ratio of 1:20, and the injection mode was manual injection. Mass spectrometry conditions: the ion source temperature was 230°C and transfer line temperature was 280°C. The ionization method was electron ionization (EI) with electron energy of 70 eV. The mass scanning range was 40–550 m z^–1^, the acquisition mode was full scan mode, and the ion source vacuum was 7.2 × 10^–7^ mTorr (Laopongsit et al., 2014). The volatile chemicals of *L. chinensis* silage were qualitatively analyzed using the NIST11 and NIST11s databases, and the relative content of each component was calculated using the peak area normalization method.

### Statistical Analysis

Two-way ANOVA and Dunnett’s multiple comparisons were performed on the chemical composition of silage using SAS 9.2, and statistical significance was at the level of *P* < 0.05 level. Bioinformatic analysis was performed using the OmicStudio tools at https://www.omicstudio.cn/tool. Tables were prepared in Excel 2010 and VIP score graph drawn with SIMCA 14.1. Pearson correlations were calculated and graphs were made in GraphPad prism 8.0.2 and R version 3.6.3.

## Results

### Fresh Characteristics

The chemical parameters and microbial compositions of *L. chinensis* before ensiling are described in Table 1. The DM content of fresh *L. chinensis* was 333.10 g/kg of FM and the contents of CP, WSC, EE, NDF, and ADF were 119.15, 50.37, 26.21, 735.50, and 404.73 g/kg DM, respectively. Microbial compositions in the *L. chinensis* for lactic acid bacteria, aerobic bacteria, coliform bacteria, and yeasts were 1.59, 2.57, 2.62, and 1.77 lg cfu/g FM, respectively, and no molds was detected.

**TABLE 1:** Chemical and microbial compositions of fresh *Leymus chinensis*.

	Items	Sample	SEM
Chemical composition	DM (g/kg FM)	333.10	2.51
	CP (g/kg DM)	119.15	0.41
	WSC (g/kg DM)	50.37	0.61
	NDF (g/kg DM)	735.50	1.14
	ADF (g/kg DM)	404.73	11.32
	EE (g/kg DM)	26.21	1.60
Microbial counts	Lactic acid bacteria (lg cfu/g FM)	1.59	0.06
	Aerobic bacteria (lg cfu/g FM)	2.57	0.42
	Coliform bacteria (lg cfu/g FM)	2.62	0.15
	Yeasts (lg cfu/g FM)	1.77	0.67
	Molds (lg cfu/g FM)	ND	ND

*FM, fresh material; DM, dry matter; CP, crude protein; WSC, water soluble carbohydrates; NDF, neutral detergent fiber; ADF, acid detergent fiber; EE, crude fat; ND, not detected; SEM, standard error of the mean.*

### Effect of Lactic Acid Bacteria Additives on Aerobic Stability During Aerobic Exposure

**FIGURE 1:**
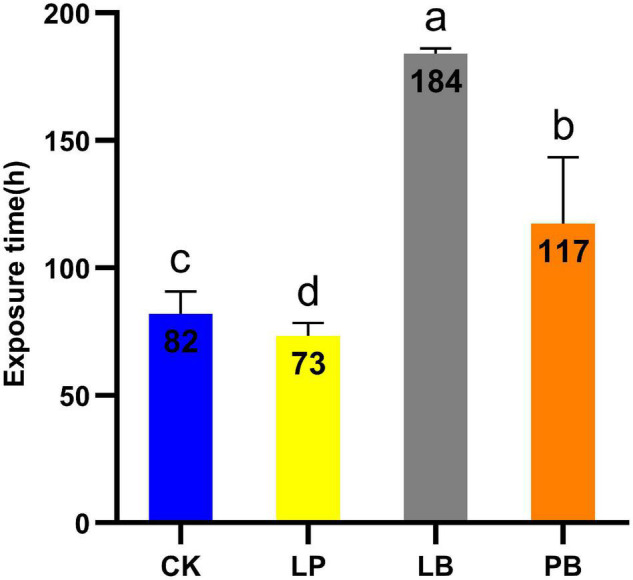
Time required to exceed room temperature 2°C during aerobic exposure of *Leymus chinensis* silage. CK, no additive control; LP, *Lactobacillus plantarum*; LB, *Lactobacillus buchneri*; PB, *Lactobacillus plantarum* + *Lactobacillus buchneri*. Different lowercase letters indicate significant differences among different treatment (*P* < 0.05).

The aerobic stability of *L. chinensis* silages is shown in Figure 1. The temperature of the CK treatment was 2°C higher than room temperature after 82 h of aerobic exposure. The temperature of the LB and PB treatments were 2°C higher than room temperature after 184 h and 117 h of aerobic exposure, respectively, which were significantly higher than that of the CK treatment (*P* < 0.05), while the aerobic stability of LP was 73 h, which was significantly lower than that of CK (*P* < 0.05). The aerobic stability of each group from high to low followed the order LB, PB, CK, and LP.

### Effects of Additives and Days of Aerobic Exposure on Chemical Composition of *Leymus chinensis* Silage

The chemical composition of *L. chinensis* silage is shown in Table 2. Both additives and days of aerobic exposure significantly affected DM, CP, WSC, and EE, but not NDF or ADF, and their interaction only significant affected WSC (*P* < 0.05). DM, CP, WSC, and EE decreased significantly with increasing days of aerobic exposure in all groups (*P* < 0.05). At 0 day of aerobic exposure, the DM of the additive group was significantly higher than that of the CK group (*P* < 0.05), but there was no significant difference among the groups at 2 and 8 days (*P* > 0.05). The CP of LP was significantly higher than other groups at 0, 4, and 6 days of aerobic exposure (*P* < 0.05), but the difference between groups at 8 days was not statistically significant (*P* > 0.05). WSC was significantly higher in LP than in other groups at 0, 2, and 4 days of aerobic exposure (*P* < 0.05), but there was no statistically significant difference between groups at 8 days (*P* > 0.05). There was no significant difference in EE among the groups at 0 day of aerobic exposure (*P* > 0.05), and the CK group was significantly lower than PB at 8 days (*P* < 0.05). NDF and ADF showed an increasing trend with aerobic exposure in each group, but the difference was not statistically significant (*P* > 0.05).

**TABLE 2:** Effects of additives and days of aerobic exposure on chemical composition of *Leymus chinensis* silage.

Items	Treatments	Days					Significance			
		0	2	4	6	8	SEM	*T*	*D*	T × D
DM (g/kg FM)	CK	434.77Ba	372.30Ab	361.17Bb	325.50Bc	307.83Ac	10.03	*	**	NS
	LP	454.83Aa	401.77Ab	376.37ABc	334.07ABd	313.37Ae				
	LB	438.83ABa	395.47Aab	391.33Ab	353.73Ab	347.23Ab				
	PB	444.73ABa	382.27Ab	370.53Bb	337.97ABc	323.47Ac				
CP (g/kg DM)	CK	149.99Ba	144.36Bab	142.88Cab	139.18Bb	130.15Ac	2.08	**	**	NS
	LP	167.44Aa	163.92Aab	158.86Aab	156.32Ab	138.57Ac				
	LB	151.60Ba	149.55Ba	149.18Ba	145.80Ba	134.34Ab				
	PB	153.88Ba	153.33ABab	151.37Bab	146.16Bab	144.05Ab				
WSC (g/kg DM)	CK	15.97Ca	12.37Cb	11.63Cbc	10.93Bbc	9.87Ac	0.77	**	**	**
	LP	22.27Aa	19.43Ab	16.57Ac	13.97Ad	11.93Ae				
	LB	17.13BCa	15.27Bb	14.67Bb	12.53ABc	11.77Ac				
	PB	18.93Ba	18.30Aa	14.00Bb	1.1.33Bc	10.40Ac				
NDF (g/kg DM)	CK	635.10	637.40	639.90	642.77	655.97	1.64	NS	NS	NS
	LP	623.33	630.37	641.70	644.77	651.70				
	LB	634.23	636.00	636.20	637.47	642.53				
	PB	630.57	635.60	634.80	640.57	643.80				
ADF (g/kg DM)	CK	379.33	381.10	386.00	389.20	388.20	1.40	NS	NS	NS
	LP	363.40	369.03	370.37	376.00	378.27				
	LB	372.80	373.70	374.40	374.87	375.13				
	PB	374.80	375.23	377.83	379.83	380.57				
EE (g/kg DM)	CK	36.03Aa	29.20ABab	27.83Ab	26.93Ab	16.00Bc	1.38	*	**	NS
	LP	40.67Aa	27.576Bb	25.87Ab	25.07Ab	22.80ABb				
	LB	39.17Aa	31.93ABab	31.60Aab	28.33Aab	24.77ABb				
	PB	39.93Aa	35.90Aab	32.37Aab	32.07Aab	28.37Ab				

FM, fresh material; DM, dry matter; CP, crude protein; WSC, water soluble carbohydrates; NDF, neutral detergent fiber; ADF, acid detergent fiber; EE, crude fat; CK, no additive control; LP, Lactobacillus plantarum; LB, Lactobacillus buchneri; PB, Lactobacillus plantarum + Lactobacillus buchneri. Different capital letters indicate significant differences among different treatments under the same silage days (P < 0.05); different lowercase letters indicate significant differences among different silage days under the same treatment (P < 0.05); no or same letter indicate not significant (P > 0.05). SEM, standard error of the mean; T, treatments; D, aerobic exposure days; T × D, interaction between treatments and Aerobic exposure days. *Significant at 0.05. **Significant at 0.01.

### Effects of Additives and Days of Aerobic Exposure on Fermentation Quality of *Leymus chinensis* Silage

The fermentation quality during aerobic exposure of *L. chinensis* silage is shown in Table 3. Additives, days of aerobic exposure and their interactions had significant effects on pH, LA, AA, PA, BA, and ammonia nitrogen content (*P* < 0.05). The pH of all groups increased significantly with increasing of aerobic exposure days (*P* < 0.05). During aerobic exposure, CK was always significantly higher than the other groups (*P* < 0.05), and LP was significantly lower than the other groups at 0 and 2 days of aerobic exposure (*P* < 0.05). LB was significantly lower than the other groups at 8 days of aerobic exposure (*P* < 0.05), and LP was significantly higher than LB and PB (*P* < 0.05). LA, AA, and PA in each group decreased significantly with increasing days of aerobic exposure (*P* < 0.05). At 0 and 2 days of aerobic exposure, LA was significantly higher in the LP group than in the other groups (*P* < 0.05), and CK was significantly lower than that of other groups at 8 days (*P* < 0.05), while there was no significant difference between additive groups (*P* > 0.05). At 0 day of aerobic exposure, AA and PA of the CK group were significantly lower than other groups, and LB was significantly higher than LP at 8 days (*P* < 0.05). BA and NH_3_-N increased significantly with increasing days of aerobic exposure in all groups (*P* < 0.05). At 0 day of aerobic exposure, BA and NH_3_-N were significantly lower in the LB group than in CK and LP (*P* < 0.05), and at 8 days of aerobic exposure, BA was significantly lower in the LB group than in the other groups (*P* < 0.05). NH_3_-N was significantly higher in the LP group than in the other groups (*P* < 0.05).

**TABLE 3:** Effects of additives and days of aerobic exposure on fermentation quality of *Leymus chinensis* silage.

Items	Treatments	Days					Significance			
		0	2	4	6	8	SEM	*T*	*D*	T × D
pH value	CK	4.60Ae	4.76Ad	4.95Ac	5.24Ab	5.47Aa	0.04	**	**	**
	LP	4.18Ce	4.37Cd	4.69Bc	4.91Bb	5.256Ba				
	LB	4.38Bd	4.49Bc	4.68Bb	4.88Ba	4.93Da				
	PB	4.32Be	4.45BCd	4.68Bc	4.93Bb	5.03Ca				
LA (g/kg DM)	CK	26.57Ca	23.92Ba	12.65Ab	12.03Ab	7.62Bc	1.61	**	**	**
	LP	53.44Aa	47.92Ab	14.54Ac	14.11Ac	10.14Ad				
	LB	29.77Ba	22.90Bb	13.80Ac	11.71Acd	9.92Ad				
	PB	32.43Ba	21.97Bb	14.39Ac	13.70Acd	10.73Ad				
AA (g/kg DM)	CK	15.95Ca	15.78Ba	13.36Bb	8.32Cc	7.25Cc	1.01	**	**	**
	LP	27.81Ba	25.48Aa	14.27Bb	10.35Bc	8.75Bc				
	LB	30.13Aa	27.00Ab	22.50Ac	13.87Ad	9.68Ae				
	PB	28.82ABa	25.96Ab	15.24Bc	10.59Bd	9.07ABe				
PA (g/kg DM)	CK	0.32Ca	0.32Ba	0.27Bb	0.17Cc	0.14Cc	0.02	**	**	**
	LP	0.56Ba	0.51Aa	0.29Bb	0.20Bc	0.17Bc				
	LB	0.60Aa	0.54Aa	0.45Ab	0.28Ac	0.20Ad				
	PB	0.58ABa	0.52Ab	0.31Bc	0.21Bd	0.18Bd				
BA (g/kg DM)	CK	0.22Ac	0.22Ac	0.3Ab	0.33Aab	0.36Aa	0.01	**	**	**
	LP	0.21Ab	0.21Ab	0.22Bb	0.34Aa	0.35Aa				
	LB	0.15Bb	0.15Bb	0.20Ba	0.22Ba	0.21Ba				
	PB	0.17Bc	0.17Bc	0.21Bb	0.34Aa	0.34Aa				
NH_3_-N (g/kg DM)	CK	0.17Ad	0.31Acd	0.48Abc	0.66Ab	0.98Ba	0.08	**	**	**
	LP	0.15ABd	0.21Ad	0.49Ac	0.84Ab	1.45Aa				
	LB	0.07Be	0.18Ad	0.31Ac	0.59Ab	0.81Ba				
	PB	0.13ABd	0.20Ad	0.41Ac	0.66Ab	0.89Ba				

pH, potential of hydrogen; LA, lactic acid; AA, acetic acid; PA, propionic acid; BA, butyric acid; NH_3_-N, ammonia nitrogen; CK, no additive control; LP, Lactobacillus plantarum; LB, Lactobacillus buchneri; PB, Lactobacillus plantarum + Lactobacillus buchneri. Different capital letters indicate significant differences among different treatments under the same silage days (P < 0.05); different lowercase letters indicate significant differences among different silage days under the same treatment (P < 0.05); no or same letter indicate not significant (P > 0.05). SEM, standard error of the mean; T, treatments; D, aerobic exposure days; T × D, interaction between treatments and Aerobic exposure days. **Significant at 0.01.

### Effect of Lactic Acid Bacteria Additives on Microbial Alpha Diversity During Aerobic Exposure

The bacterial alpha diversity during aerobic exposure of *L. chinensis* silage is shown in Table 4. Days of aerobic exposure had a significant effect on OTUs, Chao1, Shannon, and Simpson (*P* < 0.05), while additives and their interactions had no significant effect (*P* > 0.05). The OTUs, Chao1, Shannon, and Simpson increased with increasing days of aerobic exposure in all groups, and bacterial coverage in all groups was 0.99. At 0 day of aerobic exposure, the OTUs and Chao1 were higher in the CK group than in the LB and PB groups, while Shannon and Simpson were lower in the LP group than in the other groups. At 4 days of aerobic exposure, the OTUs and Chao1 were higher in the LP group than in the other groups, while Shannon and Simpson indexes were lower in the LP group than in the other groups. At 8 days of aerobic exposure, the OTUs, Chao1, Shannon, and Simpson were higher in the CK group than in the other groups.

**TABLE 4:** Effects of additives and days of aerobic exposure on bacterial alpha diversity of *Leymus chinensis* silage.

Items	Treatments	Time (d)			Significance			
		0	4	8	SEM	*T*	*D*	T × D
OTUs	CK	183.67Ab	192.00Ab	378.33Aa	12.82	NS	**	NS
	LP	184.00Aa	199.33Aa	262.67Aa				
	LB	170.67Aa	215.67Aa	260.67Aa				
	PB	166.00Aa	184.00Aa	218.00Aa				
Chao1	CK	183.97Ab	192.07Ab	378.82Aa	12.83	NS	**	NS
	LP	184.00Aa	199.51Aa	263.21Aa				
	LB	170.81Aa	215.67Aa	260.83Aa				
	PB	166.41Aa	184.03Aa	218.05Aa				
Shannon	CK	2.41Aa	2.56Aa	4.27Aa	0.13	NS	*	NS
	LP	2.14Aa	2.18Aa	2.84Ba				
	LB	2.49Aa	2.82Aa	3.03ABa				
	PB	2.28Aa	2.59Aa	2.75Ba				
Simpson	CK	0.61Aa	0.62Aa	0.84Aa	0.02	NS	*	NS
	LP	0.59Aa	0.56Aa	0.67Ba				
	LB	0.66Aa	0.67Aa	0.76ABa				
	PB	0.60Ab	0.67Aab	0.72ABa				
Coverage	CK	0.99	0.99	0.99	0.00	NS	NS	NS
	LP	0.99	0.99	0.99				
	LB	0.99	0.99	0.99				
	PB	0.99	0.99	0.99				

CK, no additive control; LP, Lactobacillus plantarum; LB, Lactobacillus buchneri; PB, Lactobacillus plantarum + Lactobacillus buchneri. Different capital letters indicate significant differences among different treatments under the same silage days (P < 0.05); different lowercase letters indicate significant differences among different silage days under the same treatment (P < 0.05); no or same letter indicate not significant (P > 0.05). SEM, standard error of the mean; T, treatments; D, aerobic exposure days; T × D, interaction between treatments and Aerobic exposure days. *Significant at 0.05. **Significant at 0.01.

The fungal alpha diversity during aerobic exposure of *L. chinensis* silage is shown in Table 5. Additives significantly affected OUTs and Chao1, and days of aerobic exposure significantly affected Shannon and Simpson indexes (*P* < 0.05), while their interactions had no significant effect (*P* > 0.05). The Shannon and Simpson indexes decreased with increasing days of aerobic exposure in all groups, and the fungal coverage in all groups was 0.99. The OTUs in the LB group showed an increasing trend followed by a decreasing trend, while the other groups showed a continuous decrease. At 0 day of aerobic exposure, the OTUs and Chao1 of the LP group were lower than other groups. At 4 days of aerobic exposure, the Shannon and Simpson indexes in the LP group were higher than those in the other groups. At 8 days of aerobic exposure, Shannon, and Simpson indexes were higher in the CK group than in the other groups.

**TABLE 5:** Effects of additives and days of aerobic exposure on fungal alpha diversity of *Leymus chinensis* silage.

Items	Treatments	Time (d)			Significance			
		0	4	8	SEM	*T*	*D*	T × D
OTUs	CK	148.00Aa	127.33Aa	125.00Aa	5.85	*	NS	NS
	LP	109.33Ba	92.33Aa	94.00Aa				
	LB	106.00Ba	153.33Aa	126.90Aa				
	PB	171.33Aa	127.54Aa	96.70Aa				
Chao1	CK	112.10Aa	92.62Aa	102.83Aa	5.86	*	NS	NS
	LP	106.08Ba	155.37Aa	109.65Aa				
	LB	171.50Ba	146.32Aa	260.83Aa				
	PB	166.41Aa	184.03Aa	218.05Aa				
Shannon	CK	3.95Aa	3.67Aa	3.21Aa	0.16	NS	**	NS
	LP	4.35Aa	4.17Aa	2.6Ab				
	LB	4.16Aa	3.75Aab	2.59Ab				
	PB	4.33Aa	3.83Aa	1.97Ab				
Simpson	CK	0.85Aa	0.85Aa	0.72Aa	0.03	NS	**	NS
	LP	0.91Aa	0.89Aa	0.63Ab				
	LB	0.89Aa	0.83Aa	0.59Aa				
	PB	0.89Aa	0.83Aa	0.47Ab				
Coverage	CK	0.99	0.99	0.99	0.00	NS	NS	NS
	LP	0.99	0.99	0.99				
	LB	0.99	0.99	0.99				
	PB	0.99	0.99	0.99				

CK, no additive control; LP, Lactobacillus plantarum; LB, Lactobacillus buchneri; PB, Lactobacillus plantarum + Lactobacillus buchneri. Different capital letters indicate significant differences among different treatments under the same silage days (P < 0.05); different lowercase letters indicate significant differences among different silage days under the same treatment (P < 0.05); no or same letter indicate not significant (P > 0.05). SEM, standard error of the mean; T, treatments; D, aerobic exposure days; T × D, interaction between treatments and Aerobic exposure days. *Significant at 0.05. **Significant at 0.01.

### Effect of Lactic Acid Bacteria Additives on Microbial Community Dynamics During Aerobic Exposure

**FIGURE 2:**
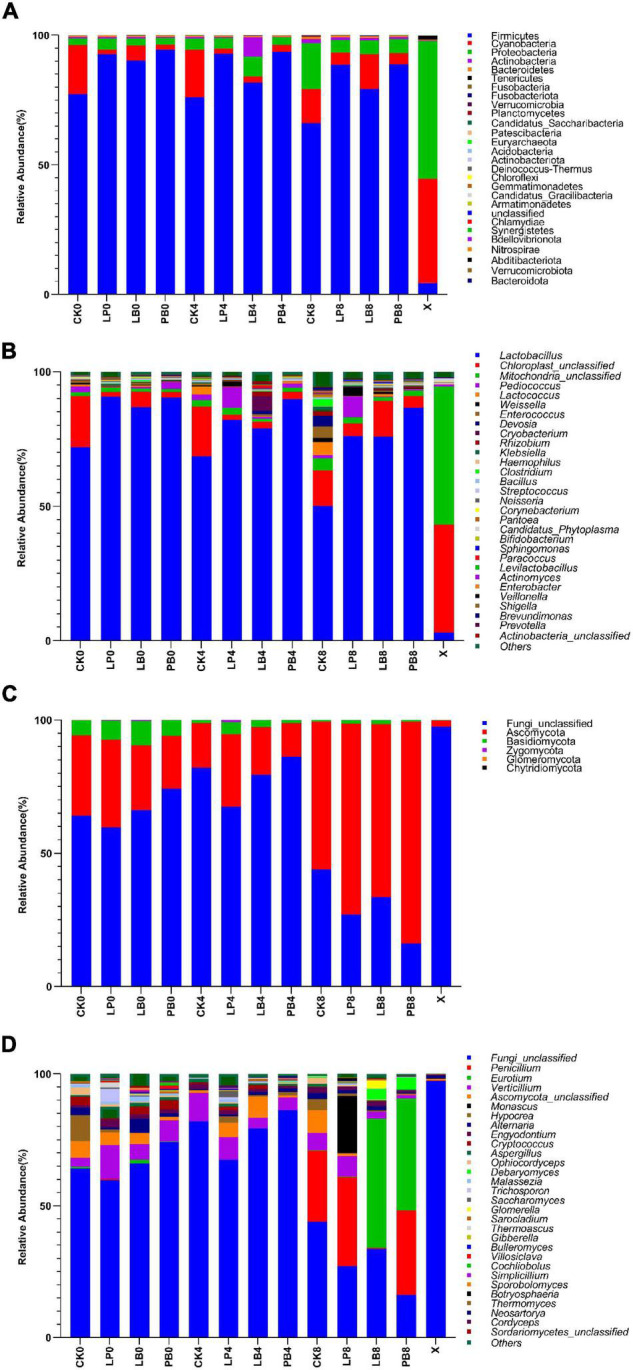
Microbial community at the phylum-level **(A,C)** and genus-level **(B,D)** abundance changes during aerobic exposure of silage. CK, no additive control; LP, *Lactobacillus plantarum*; LB, *Lactobacillus buchneri*; PB, *Lactobacillus plantarum* + *Lactobacillus buchneri*; X, fresh sample.

This study determined phylum-level and genus-level changes in the microbial community of *L. chinensis* silage during aerobic exposure (Figure 2). The phylum-level changes in bacterial communities are shown in Figure 2A. Compared to fresh samples, Firmicutes replaced Cyanobacteria and Proteobacteria as the dominant phyla after ensilement. Firmicutes, Cyanobacteria, and Proteobacteria were the main phyla in each group. The abundance of Firmicutes gradually decreased with the extension of aerobic exposure time but remained at a high level, and the abundance of Firmicutes in the additive groups was higher than the level of the control group. The abundance of Cyanobacteria in the additive groups increased during 8 days of aerobic exposure. The abundance of Proteobacteria gradually increased with increasing aerobic exposure time, but the degree of change was significantly higher in the CK treatment than in the other groups.

The genus-level changes of the bacterial community are shown in Figure 2B. The abundance of *Lactobacillus* in all groups showed a downward trend with increasing aerobic exposure time, but the bacterial community in the CK group was significantly different from that in the additive groups. The abundance of *Lactobacillus* in the CK group decreased from 70 to 50%, while the abundance of miscellaneous bacteria such as *Enterococcus*, *Devosella*, and *Klebsiella* increased significantly. The abundance of *Lactobacillus* in the additive groups decreased from 90 to 70% and remained at a relatively high level. The abundance of *Pediococcus* and *Weissella* in the LP treatment and the abundance of *Klebsiella* in each group increased during aerobic exposure. The level of bacterial genera in the CK group changed to a greater extent after aerobic exposure than in the additive groups, and the abundance of miscellaneous bacteria in each group increased significantly.

The phylum-level changes of the fungal community are shown in Figure 2C. Compared to fresh samples, Ascomycota and Basidiomycota became the dominant phylum after ensilement. At 4 days of aerobic exposure, the abundances of Ascomycota and Basidiomycota in the LP group were higher than in other groups. At 8 days, the abundance of Ascomycota increased significantly in all groups, with greater changes in the LB and PB groups.

The genus-level changes of the fungal community are shown in Figure 2D. Compared to fresh samples, the fungal genus level composition of each group changed significantly during aerobic exposure, and the abundance of *Penicillium*, *Eurotium*, and *Monascus* in each group increased significantly. The abundance of *Verticillium* gradually decreased with extension of aerobic exposure time. At 8 days of aerobic exposure, the abundance of *Monascus* increased significantly in the LP group, the abundance of *Eurotium* and *Debaryomyces* increased significantly in the LB and PB groups, and the abundance of *Penicillium* increased significantly increased in all groups except the LB group.

### Effect of Lactic Acid Bacteria Additives on Volatile Chemicals During Aerobic Exposure

**FIGURE 3:**
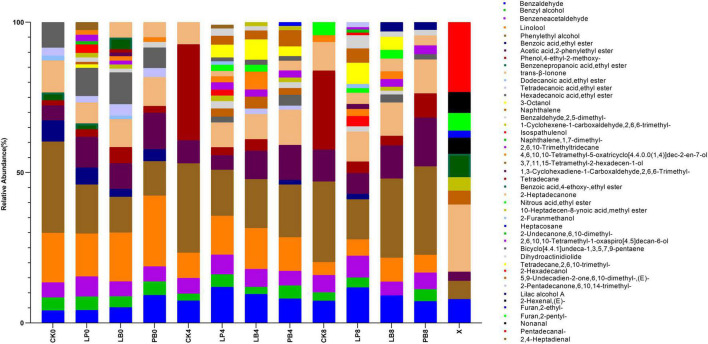
Changes in the abundance of volatile chemicals during aerobic exposure of silage. CK, no additive control; LP, *Lactobacillus plantarum*; LB, *Lactobacillus buchneri*; PB, *Lactobacillus plantarum* + *Lactobacillus buchneri*; X, fresh sample.

This study investigated the changes in the abundance of volatile chemicals during aerobic exposure of *L. chinensis* silage (Figure 3). A total of 45 substances were detected (Supplementary Table 1), including 10 esters, 10 alcohols, 9 aldehydes, 6 ketones, 5 alkanes, 3 heterocycles, 1 alkene, and 1 phenol. Esters, alcohols, and aldehydes were the main volatile chemicals during aerobic exposure of *L. chinensis* silage. Compared to fresh samples, the dominant volatile chemicals after ensilement changed from Pentadecanal (C_15_H_30_O) and *Trans-*β-Ionone (C_13_H_20_O) to Phenylethyl alcohol (C_8_H_10_O), Linalool (C_10_H_18_O) and Acetic acid, 2-phenylethyl ester (C_10_H_12_O_2_). During aerobic exposure, the abundances of C_10_H_18_O continued to decrease in all groups, but the abundance of C_8_H_10_O remained high, and the abundance of Phenol, 4-ethyl-2-methoxy- (C_9_H_12_O_2_) increased significantly in the CK group. The variety of volatile chemicals, such as Isospathulenol (C_15_H_24_O), Benzaldehyde (C_7_H_6_O), 2-Furancarbinol (C_5_H_6_O_2_) and Naphthalene (C_10_H_8_), was higher in the additive group than in the control group and increased significantly in the LP group with increasing days of aerobic exposure. *Trans-*β-Ionone (C_13_H_20_O) was always present during the experiment.

**FIGURE 4:**
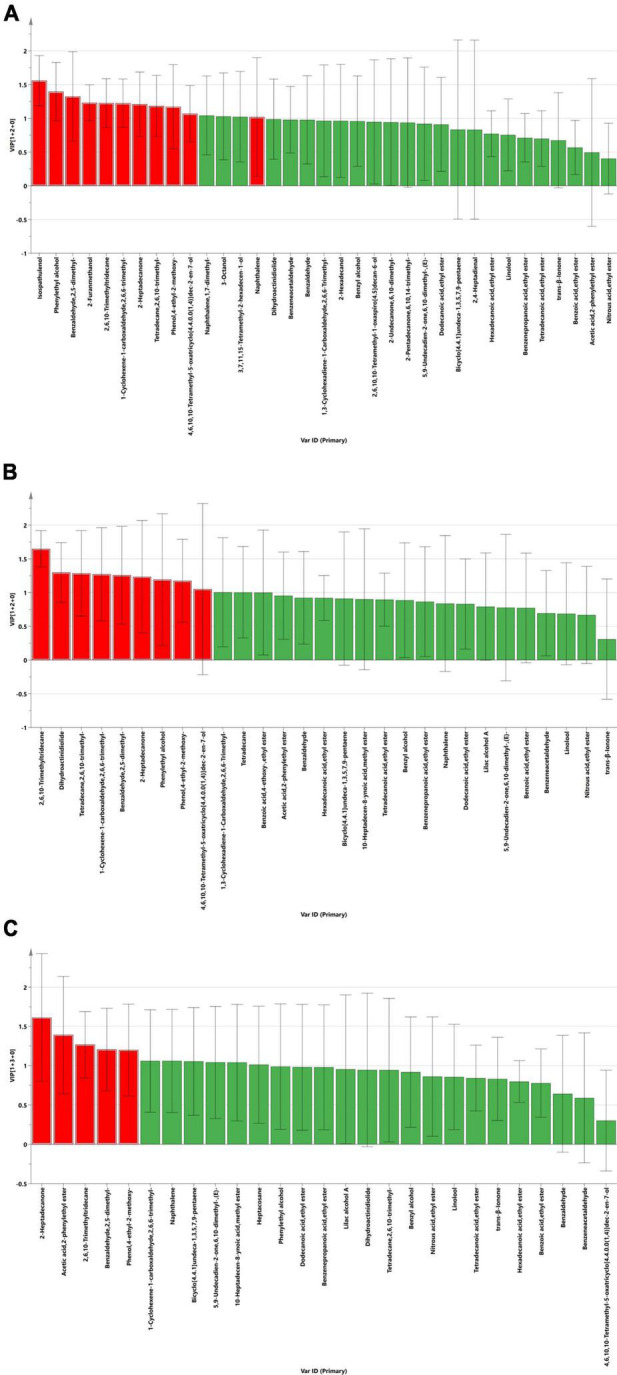
Differences in volatile chemicals between silages treated with LP **(A)**, LB **(B)**, and PB **(C)** compared to silage treated with CK. Volatile chemicals with VIP > 1.0 and *P* < 0.05 were marked as red.

The VIP score is a variable that predicts importance and represents the difference between groups. In this study, VIP scores were obtained by least squares discriminant analysis, for which it is generally accepted that there is a significant difference between groups when VIP > 1.0. Independent samples *t*-tests for volatile components with VIP scores greater than 1, combined with VIP > 1.0 and *P* < 0.05, were used to screen out differential volatile components (Supplementary Table 2). The differences in volatile chemicals in the LP (A), LB (B), and PB (C) groups during aerobic exposure compared to the *L. chinensis* silage in the CK group are shown in Figure 4. The differential volatile chemicals in the LP and CK groups included Isospathulenol (C_15_H_24_O), Phenylethyl alcohol (C_8_H_10_O), Benzaldehyde, 2,5-dimethyl- (C_9_H_10_O), 2-Furanmethanol (C_5_H_6_O_2_), 2,6,10-Trimethyltridecane (C_16_H_34_), 1-Cyclohexene-1-carboxaldehyde, 2,6,6-trimethyl- (C_10_H_16_O), 2-Heptadecanone (C_17_H_34_O), Tetradecane, 2,6,10-trimethyl- (C_17_H_36_), Phenol, 4-ethyl-2-methoxy- (C_9_H_12_O_2_), 5-Oxatricyclo[4.4.0.01,4]dec-2-en-7-ol, 4,6,10,10- tetramethyl-, (1R,4R,6S,7R)-rel- (9CI) (C_13_H_20_O_2_) and Naphthalene (C_10_H_8_). In the LB and CK groups, differential volatile chemicals included 2,6,10-Trimethyltridecane (C_16_H_34_), Dihydroactinidiolide (C_11_H_16_O_2_), Tetradecane, 2,6,10-trimethyl- (C_17_H_36_), 1-Cyclohexene-1-carboxaldehyde, 2,6,6-trimethyl- (C_10_H_16_O), Benzaldehyde, 2,5-dimethyl- (C_9_H_10_O), 2-Heptadecanone (C_17_H_34_O), Phenylethyl alcohol (C_8_H_10_O), Phenol, 4-ethyl-2-methoxy- (C_9_H_12_O_2_) and 5-Oxatricyclo[4.4.0.01,4]dec-2-en-7-ol, 4,6,10,10- tetramethyl-, (1R,4R,6S,7R)-rel- (9CI) (C_13_H_20_O_2_). In the PB and CK groups, differential volatile chemicals included 2-Heptadecanone (C_17_H_34_O), Acetic acid, 2-phenyl ethylester (C_10_H_12_O_2_), 2,6,10-Trimethyltridecane (C_16_H_34_), Benzaldehyde, 2,5-dimethyl- (C_9_H_10_O), and Phenol, 4-ethyl-2-methoxy- (C_9_H_12_O_2_).

### Correlation of Microbial Genera Level With Silage Quality and Differential Volatile Chemicals During Aerobic Exposure

**FIGURE 5:**
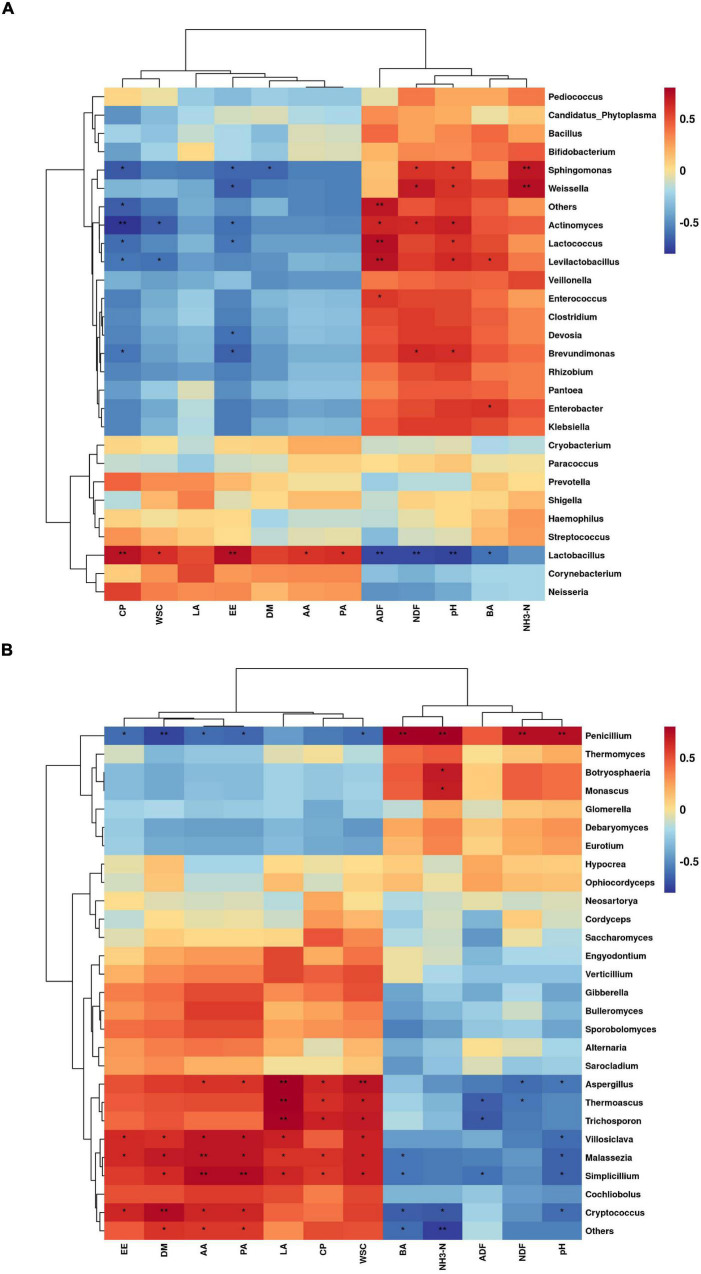
Correlation of bacterial genera level **(A)** and fungal genera level **(B)** with silage quality during aerobic exposure of silage (*P < 0.05; **P < 0.01).

The correlations between bacterial genus levels and silage quality during aerobic exposure are shown in Figure 5A. *Lactobacillus* was extremely significantly positively correlated with CP and EE (*P* < 0.01), and significantly positively correlated with WSC, AA, and PA (*P* < 0.05), but was significantly negatively correlated with pH, ADF, NDF, and BA (*P* < 0.05). By contrast, *Sphingomonas* and *Weissella* were extremely significantly positively correlated with NH_3_-H (*P* < 0.01), and significantly positively correlated with pH and NDF (*P* < 0.05). *Sphingomonas* was significantly negatively correlated with DM, CP, and EE (*P* < 0.05), and *Weissella* was significantly negatively correlated with EE (*P* < 0.05). *Actinomycetes* was extremely significantly negatively correlated with CP (*P* < 0.01) and significantly negatively correlated with WSC and EE (*P* < 0.05), but significantly positively correlated with CP, WSC, and EE (*P* < 0.05). *Lactococcus* and *Levilactobacillus* were extremely significantly positively correlated with ADF (*P* < 0.01), and were significantly positively correlated with pH (*P* < 0.05), while significantly negatively correlated with CP and EE (*P* < 0.05). *Brevundimonas* was significantly positively correlated with pH and NDF (*P* < 0.05), and significantly negatively correlated with CP and EE (*P* < 0.05).

The correlations between fungal levels and silage quality during aerobic exposure are shown in Figure 5B. *Penicillium* was extremely significantly positively correlated with pH, NDF, NH_3_-N, and BA (*P* < 0.01), but extremely significantly negatively correlated with DM (*P* < 0.01) and significantly negatively correlated with WSC, EE, AA, and PA (*P* < 0.05). *Botryosphaeria* and *Monascus* were significantly positively correlated with NH_3_-N (*P* < 0.05). *Aspergillus*, *Thermoascus*, and *Trichosporon* were extremely significantly positively correlated with LA (*P* < 0.01) and significantly positively correlated with CP and WSC (*P* < 0.05). Fungi, such as *Cryptococcus*, *Malassezia*, *Simplicillium*, were positively correlated with DM, WSC, and EE (*P* < 0.05) and negatively correlated with pH and BA (*P* < 0.05).

**FIGURE 6:**
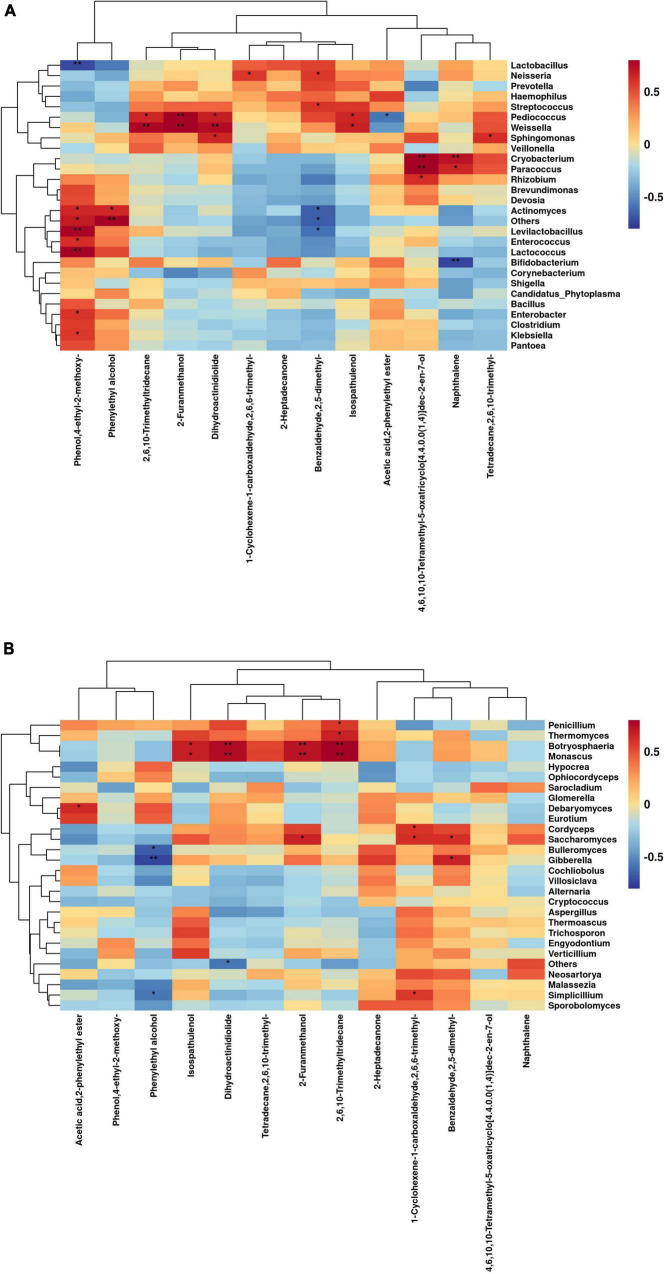
Correlation of bacterial **(A)** and fungal **(B)** genus levels with volatile chemicals during aerobic exposure of silage (*P < 0.05; **P < 0.01).

The correlations between bacterial genus levels and differential volatile chemicals during aerobic exposure are shown in Figure 6A. *Actinomyces* was significantly positively correlated with Phenylethyl alcohol (C_8_H_10_O) and Phenol, 4-ethyl-2-methoxy- (C_9_H_12_O_2_) (*P* < 0.05), but significantly negatively correlated with Benzaldehyde, 2,5-dimethyl-PA (C_9_H_10_O) (*P* < 0.05). *Levilactobacillus* and *Lactococcus* were extremely significantly positively correlated with Phenol, 4-ethyl-2-methoxy- (C_9_H_12_O_2_) (*P* < 0.01). *Cryobacterium* was extremely significantly positively correlated with Naphthalene (C_10_H_8_) (*P* < 0.01), while *Bifidobacterium* was extremely significantly negatively correlated with it (*P* < 0.01). *Pediococcus* was extremely significantly positively correlated with 2-Furanmethanol (C_5_H_6_O_2_) (*P* < 0.01), and was significantly positively correlated with Isospathulenol (C_15_H_24_O), Dihydroactinidiolide (C_11_H_16_O_2_) and 2,6,10-Trimethyltridecane (C_16_H_34_) (*P* < 0.05), but was significantly negatively correlated with Acetic acid, 2-phenylethyl ester (C_10_H_12_O_2_) (*P* < 0.05). *Weissella* was extremely significantly positively correlated with 2-Furanmethanol (C_5_H_6_O_2_), Dihydroactinidiolide (C_11_H_16_O_2_), and 2,6,10-Trimethyltridecane (C_16_H_34_) (*P* < 0.01) and significantly positively correlated with Isospathulenol (C_15_H_24_O) (*P* < 0.05). *Lactobacillus* was extremely significantly negatively correlated with Phenol, 4-ethyl-2-methoxy- (C_9_H_12_O_2_) (*P* < 0.01).

The correlation between fungal genus levels and differential volatile chemicals during aerobic exposure are shown in Figure 6B. *Botryosphaeria* and *Monascus* were extremely significantly positively correlated with 2-Furanmethanol (C_5_H_6_O_2_), 2,6,10-Trimethyltridecane (C_16_H_34_), and Dihydroactinidiolide (C_11_H_16_O_2_) (*P* < 0.01), and significantly positively correlated with Isospathulenol (C_15_H_24_O) (*P* < 0.05). *Gibberella*, *Bulleromyces*, and *Simplicillium* were significantly negatively correlated with Phenylethyl alcohol (C_8_H_10_O) (*P* < 0.05). *Cordyceps*, *Saccharomyces*, and *Simplicillium* were significantly positively correlated with 1-Cyclohexene-1-carboxaldehyde, 2,6,6-trimethyl- (C_10_H_16_O) (*P* < 0.05), and *Saccharomyces* was significantly positively correlated with 2-Furanmethanol (C_5_H_6_O_2_) and Benzaldehyde, 2,5-dimethyl-PA (C_9_H_10_O) (*P* < 0.05).

## Discussion

The lactic acid bacteria count and water-soluble carbohydrate content of raw materials are important considerations when making silage. Silage is more likely to be successful when the lactic acid bacteria content in raw materials exceeds 10^5^ cfu/g and the WSC content exceeds 50 g/kg DM (Cai et al., 1998). In this study, the raw material had a low number of lactic acid bacteria and a high number of coliforms, with a WSC of 50.37 g/kg DM, so direct ensilement would not be likely to succeed, making it necessary to improve the ensilement effect by using a lactic acid bacteria additive.

The silage undergoes aerobic exposure during collection, during which the LB group had the best aerobic stability and was significantly more stable than the other groups, which indicates that the presence of *Lactobacillus buchneri* could effectively improve the aerobic stability of the silage. Similarly, Jatkauskas et al. (2018) and da Silva et al. (2019) showed that *Lactobacillus buchneri* could improve the aerobic stability of silage through the production of acetic acid. The LP group had the worst aerobic stability, which could be due to the yeasts using lactic acid produced by *Lactobacillus plantarum* to generate heat during growth and reproduction (Ranjit and Kung, 2000).

The analysis of quality changes during aerobic exposure showed that DM decreased significantly in all groups, but the loss of DM was significantly lower in the LB group than in the other groups, which was due to the fact that *Lactobacillus buchneri* effectively reduced the loss of DM during aerobic exposure by inhibiting microbial activity, which was similar to the findings of Faria et al. (2020). CP decreased significantly in all groups during aerobic exposure, and the decrease in CP accelerated with increasing pH, which is consistent with the findings of Ni et al. (2017). However, CP levels were higher in the LP group than in the other groups due to the addition of *Lactobacillus plantarum* to produce a large amount of lactic acid, which rapidly decreased the pH and inhibited the plant protein hydrolase activity of silage. WSC was low in all groups, but was consistently higher in the LP group than in the other groups, due to the conversion of starch in *L. chinensis* into soluble carbohydrates by amylase produced by *Lactobacillus plantarum*, which is in agreement with the results of Etoa et al. (2005). EE decreased significantly in all groups, but was higher in the LB and PB groups than in the other two groups due to the inhibition capacity of acetic acid which reduced the breakdown of crude fat by harmful bacteria and also hindered the fat oxidation process, which is similar to the findings of Amiri et al. (2021). The pH is an important indicator of silage quality. The pH of the additive groups was consistently lower than the control group during aerobic exposure, and the pH increased significantly in all treatment groups, but the CK and LP groups increased faster than the other two groups. These results are consistent with the findings of Liu et al. (2019). The lactate content of the CK and LP groups decreased significantly during aerobic exposure, which was due to the breakdown and utilization of lactate by microorganisms such as yeasts and aerobic bacteria, resulting in a significant increase in pH. The content of acetic acid and propionic acid decreased significantly with time in all groups, but the LB and PB groups were always higher than the other two groups because the addition of *Lactobacillus buchneri* could increase the content of acetic acid and propionic acid in silage, thus improving its aerobic stability, preventing the growth and reproduction of harmful microorganisms, and slowing down the increase in pH. As the aerobic exposure time increased, the butyric acid and ammonia nitrogen content increased significantly in all groups, but the LP and PB groups were always higher than the LB group. This was due to the poor inhibitory effect of *Lactobacillus plantarum* on the growth activity of yeasts and molds, resulting in the breakdown of some proteins and soluble carbohydrates to BA and NH_3_-N, as was also reported by Mugabe et al. (2020).

The growth and reproduction of harmful bacteria is the main factor leading to aerobic spoilage, and the analysis of microbial diversity showed that the diversity of bacterial species in each treatment tended to increase during aerobic exposure. A study by Guan et al. (2020) showed that as the duration of aerobic exposure increased, lactic acid bacteria lost their dominance, and aerobic bacteria and bacterial diversity increased, which is consistent with the results of this study. The OTUs and Chao1 values of bacteria and fungi in the CK group were higher than those in the LP and PB groups at 0 day of aerobic exposure, indicating that the addition of lactic acid bacteria could effectively inhibit the miscellaneous bacteria during silage, which is consistent with the findings of Wang et al. (2021). After 4 days of aerobic exposure, the OTUs, Chao 1, Shannon, and Simpson indices of fungi decreased in other groups, while the LP group significantly increased, indicating that the LP group was less effective in inhibiting the miscellaneous bacteria during aerobic exposure, which is similar to results reported by others (Wang et al., 2020; Xie et al., 2020). This may be due to the fact that lactic acid produced by *Lactobacillus plantarum* during aerobic exposure provides a substrate for yeasts growth and reproduction (Keshri et al., 2018). The fungal diversity in the LB group was consistently lower than in the other groups during aerobic exposure because the heterogeneous fermentation of *Lactobacillus buchneri* produced acetic and propionic acids that had a stronger inhibitory effect on the fungi, as was also reported by Guo et al. (2018).

The analysis of microbial abundance changes showed that, compared to fresh samples Firmicutes replaced Cyanobacteria and Proteobacteria as the dominant phylum of bacteria after fermentation, which is consistent with the results of Lin et al. (2021). The abundance of the Firmicutes gradually decreased with the extension of aerobic exposure time, but remained at a high level, and the abundance of Firmicutes in the additive groups was higher than in the control group, which may be related to the parthenogenetic anaerobic bacteria in the Firmicutes, as was also shown by Liu et al. (2019). The abundance of Proteobacteria gradually increased in the CK group, which may be due to competition between the Firmicutes and Proteobacteria during ensilement (He et al., 2020; Yuan et al., 2020). The abundance of miscellaneous bacteria such as *Enterococcus*, *Devosella*, and *Klebsiella* significantly increased in the CK group due to the increase in pH that promoted their growth and accelerated the deterioration of silage (Li et al., 2017; Shah et al., 2020). The abundance of *Lactobacillus* in the additive group was maintained at a high level, which may be due to the parthenogenic anaerobic nature of some lactic acid bacteria (Keshri et al., 2018; Guan et al., 2020), thus delaying the decline in the abundance of *Lactobacillus* in the additive group. Compared to fresh samples, Ascomycota and Basidiomycota became the dominant fungal phyla after ensilement, which is consistent with the findings of Romero et al. (2017). The higher abundance of Ascomycota and Basidiomycota in the LP group than other groups may be related to the poor aerobic stability of the LP group. The results of Bai et al. (2020) showed that Ascomycota and Basidiomycota are common fungal phyla in silage and their abundance increased significantly after aerobic exposure. During aerobic exposure, yeasts abundance was higher in LP than in other groups. Numerous studies have shown that lactic acid assimilating yeasts are associated with poor aerobic stability of LP treatments (Filya and Sucu, 2007; Liu et al., 2018). Also, the abundance of *Penicillium* was significantly increased in all groups except the LB group. *Penicillium* belongs to the phylum Ascomycota and lives in a saprophytic manner, feeding on decaying fruits, vegetables, meat and moist organic matter, and is a microorganism that causes spoilage of silage (Duniere et al., 2017). The abundance of *Monascus* in LP was significantly higher than in the other groups, and *Monascus* can use a variety of carbohydrates and acids, such as starch, glucose, and lactic acid, as carbon sources and produce a variety of enzymes to degrade organic matter, which is associated with aerobic spoilage (Cai et al., 2021b). Acetic acid and propionic acid produced by LB groups inhibited this fungus and improved aerobic stability. The results of this study showed that the addition of *Lactobacillus plantarum* led to an increase in the abundance of harmful microorganisms during aerobic exposure of silage, while the addition of *Lactobacillus buchneri* better inhibited their growth and multiplication for a period.

The deterioration of silage produces unpleasant odors, and by analyzing the volatile chemicals, this study found that esters, alcohols and aldehydes were the main volatile chemicals in silage, which is consistent with the findings of Weiss et al. (2016). The dominant volatile chemicals after silage were Phenylethyl alcohol (C_8_H_10_O), Linalool (C_10_H_18_O), and Acetic acid, 2-phenylethyl ester (C_10_H_12_O_2_). Among them, Phenylethyl alcohol has a sweet rose fragrance, Linalool has the fragrance of lily of the valley, and Acetic acid, 2-phenylethyl ester has a slight floral fragrance. These chemicals are commonly used in perfumes and fragrances, indicating that the odor of *L. chinensis* can be improved by ensilement. During aerobic exposure, the abundance of C_8_H_10_O remained high, which may be related to the production of alcohols metabolized by the heterobacteria during aerobic exposure. The abundance of Phenol, 4-ethyl-2-methoxy- (C_9_H_12_O_2_) was significantly higher in the CK group but lower in the additive groups, which is consistent with the finding for straw silage by Zhang et al. (2021). According to the abundance map and VIP score map, the types of volatile chemicals in the LP group and the differences in volatile chemicals from the CK group were significantly higher than those in other groups, which is possibly related to the growth and reproduction of microorganisms using lactic acid and soluble sugars as substrates, metabolizing a variety of volatile chemicals (Kung et al., 2018). Among them, Isospathulenol (C_15_H_24_O) is a volatile oil with a yellowish color and strong odor, found in Salvia by Bahadori et al. (2017). Benzaldehyde (C_7_H_6_O) and Benzaldehyde, 2,5-dimethyl- (C_9_H_10_O) have a bitter almond odor and are easily oxidized to benzoic acid in air and have the odor of benzene or formaldehyde. 2-Furanmethanol (C_5_H_6_O_2_) has a bitter and pungent odor and Naphthalene (C_10_H_8_) has a camphoraceous odor and is toxic (Marr et al., 2006; Kang and Baek, 2014; Patocka and Kuca, 2014). *Trans-*β-Ionone (C_13_H_20_O) was present throughout the experiment and is common in plants, which may be related to the odor of *L. chinensis* itself (Paparella et al., 2021). The results demonstrated that the addition of *Lactobacillus plantarum* resulted in the production of more volatile chemicals during aerobic exposure of *L. chinensis* silage, which may seriously affect livestock feed intake and silage palatability and endanger livestock health, and further studies are needed to demonstrate the metabolic pathways of these volatile chemicals.

To identify the microorganisms that cause deterioration during aerobic exposure, correlations between quality and volatile chemicals with microorganisms were analyzed. In this study, *Lactobacillus* showed significant positive correlations with CP, WSC, EE, AA, and PA, as well as with pH, ADF, NDF, and BA, indicating that the higher the abundance of lactic acid bacteria, the better the quality of the silage, which is consistent with the findings of Guo et al. (2021). Among several bacteria associated with quality decline, *Sphingomonas* can be used for the biodegradation of aromatic compounds and is a harmful bacterium affecting fermentation that affects fermentation, as it leads to elevated pH and ammonia nitrogen through acid and protein breakdown (Ni et al., 2017). *Actinomycetes* are usually present in neutral or slightly alkaline environments and degrade organic matter such as proteins and soluble sugars, reducing the quality of silage (George et al., 2012). *Weissella*, *Lactococcus*, and *Levilactobacillus* are facultative anaerobic bacteria that compete with *Lactobacillus* in the middle and late fermentation stages, and their abundance increases with the decrease of *Lactobacillus* after aerobic exposure, and increase with increasing pH and ADF (Fessard and Remize, 2017). *Brevundimonas* is an aerobic microorganism that acts as a degrading bacterium and may be associated with the breakdown of nutrients such as proteins and crude fats (Wang et al., 2022). *Penicillium* showed a highly significant positive correlation with pH, NDF, NH_3_-N, and BA, indicating that higher abundances of *Penicillium* were associated with poorer silage quality, *Penicillium* was the dominant genus during silage and aerobic exposure, and is associated with mycotoxins and quality deterioration (Del Palacio et al., 2016). *Botryosphaeria* and *Monascus* were significantly positively correlated with NH_3_-N, which indicates that they may be involved in the synthesis of ammonia nitrogen. Among them, *Botryosphaeria* is an important pathogen of fruit ulcer disease, which is widely distributed and harmful, and may be associated with aerobic spoilage of silage (Yuan et al., 2021). *Monascus* is an acid-tolerant saprophytic fungus that can tolerate high lactic acid during ensilement and grows mainly under aerobic conditions (Sun et al., 2022). Fungi such as *Aspergillus*, *Thermoascus*, and *Trichosporon* showed significant positive correlations with LA, CP, and WSC, while *Cryptococcus*, *Malassezia*, and *Simplicillium* showed positive correlations with DM, WSC, and EE, due to their rapid decrease in abundance during aerobic exposure, which resulted the same trend with nutrient decline. By contrast, the increased abundance of fungi, such as *Penicillium*, *Monascus*, *Eurotium*, and *Debaryomyces*, may be due to the competitive relationship between them. These fungi may be key microbial genera affecting silage quality during aerobic exposure, and their metabolic processes require further study.

In this study, *Lactobacillus* was significantly negatively associated with C_9_H_12_O_2_, while *Actinomyces* was significantly positively associated with it, which was due to *Lactobacillus* fermentation mainly producing acids and inhibiting the metabolism of other microorganisms (Muck, 2010). Zhang et al. (2021) showed that the addition of *Lactobacillus plantarum* inhibited the production of C_9_H_12_O_2_, and Gupta et al. (2015) found that the metabolites of *Actinomyces* promote sucrose fermentation and phenethyl alcohol production, which may explain their relevance. *Weissella* and *Pediococcus* showed significant positive correlations with C_5_H_6_O_2_, C_15_H_24_O, and C_11_H_16_O_2_. Among these compounds, C_5_H_6_O_2_ has a bitter and spicy taste, C_15_H_24_O has a strong woody odor, and C_11_H_16_O_2_ has been associated with the formation of woody aroma. *Weissella* is associated with food spoilage, producing slime and odor, and is commonly found in meat and fermented foods (Takahashi et al., 2021). *Pediococcus* can use carbohydrates to produce amylase and lipase, which break down nutrients to produce volatile chemicals and are associated with the formation of biogenic amines (Feng et al., 2021). Xu et al. (2022) showed that *Pediococcus* was associated with beer spoilage, producing odor and causing turbidity in beer, and the facultative anaerobic bacteria in *Weissella* and *Pediococcus* may be associated with the production of these odors (Cai et al., 2021a). *Bifidobacterium* showed a significant negative correlation with C_10_H_8_ due to the inhibitory effect of *Bifidobacterium* as a probiotic (Battisti et al., 2015). By contrast, *Cryobacterium* may be associated with the production of toxic and odorous substances. *Botryosphaeria* and *Monascus* showed significant positive correlations with C_5_H_6_O_2_, C_15_H_24_O, and C_11_H_16_O_2_, implying that these may be among the fungal genera that produce strong odors upon aerobic exposure. *Saccharomyces*, *Gibberella*, and *Simplicillium* were positively associated with C_5_H_6_O_2_, C_10_H_16_O, and C_9_H_10_O, which are the main microorganisms that cause aerobic spoilage (Nair et al., 2019), and C_10_H_16_O is an important intermediate for the synthesis of terpenoids such as spices and carotenoids, and terpenes are common among volatile components (Yang et al., 2021). These may be key microbial genera affecting volatile chemicals in silage during aerobic exposure, and further study of their metabolic processes would help to better define the relationship between microbes and volatile chemicals.

## Conclusion

This study showed that the DM, CP, WSC, and LA content decreased and pH, BA, and NH_3_-N increased of *L. chinensis* silage with increasing aerobic exposure time. The use of *Lactobacillus plantarum* increased the content of WSC and LA in the silage, but promoted the growth of harmful microorganisms during aerobic exposure, such as *Penicillium* and *Monascus*, and produced strong volatile chemicals such as Isospathulenol and 2-Furanmethanol. The use of *Lactobacillus buchneri* improved aerobic stability and inhibited the growth of *Penicillium* and *Monascus*. The effect of these two additives combined was intermediate between that of each additive alone. From the correlation analysis, it was concluded that *Actinomyces* and *Sphingomonas* among bacteria and *Penicillium* and *Monascus* among fungi were associated with aerobic spoilage. *Weissella* and *Pediococcus* among bacteria and *Botryosphaeria* and *Monascus* among fungi were associated with strong odors. In conclusion, inoculation with LB improved the quality of silage during aerobic exposure, while inoculation with LP accelerated aerobic degradation, the metabolic process of which needs further study.

## Data Availability Statement

The data presented in this study can be found in online repositories. The names of the repository/repositories and accession number(s) can be found below: https://www.ncbi.nlm.nih.gov/, PRJNA854793.

## Author Contributions

YiL designed the study and performed the experiments. YiL, YuL, and QL performed the data analysis and wrote the manuscript. SD and LS revised the manuscript. TL and MH edited the language. GG and ZW contributed to acquisition, review, and editing. YJ contributed to conceptualization and funding acquisition. All authors contributed to the article and approved the submitted version.

## Conflict of Interest

The authors declare that the research was conducted in the absence of any commercial or financial relationships that could be construed as a potential conflict of interest.

## Publisher’s Note

All claims expressed in this article are solely those of the authors and do not necessarily represent those of their affiliated organizations, or those of the publisher, the editors and the reviewers. Any product that may be evaluated in this article, or claim that may be made by its manufacturer, is not guaranteed or endorsed by the publisher.
