# Supplementary material for: Effects of Lactic Acid Bacteria Additives on the Quality, Volatile Chemicals and Microbial Community of Leymus chinensis Silage During Aerobic Exposure
Source: Front Microbiol. 2022 Sep 2;13:938153. doi: 10.3389/fmicb.2022.938153 (PMC9478463; doi:10.3389/fmicb.2022.938153)
Supplement: Supplementary file 1 [file Table_1.DOC]

S 1 Volatile chemical test results of *Leymus chinensis* silage.

| SN | RT | CAS | Compound | Chemical formula | Relative content (%) | | | | | | | | | | | | |
| --- | --- | --- | --- | --- | --- | --- | --- | --- | --- | --- | --- | --- | --- | --- | --- | --- | --- |
|  |  |  |  |  | CK0 | LP0 | LB0 | PB0 | CK4 | LP4 | LB4 | PB4 | CK8 | LP8 | LB8 | PB8 | X |
| 1 | 2.232 | 109-95-5 | Nitrous acid,ethyl ester | C_2_H_5_NO_2_ | 0.00 | 0.00 | 0.00 | 0.00 | 0.00 | 2.06 | 2.31 | 0.00 | 4.37 | 1.54 | 2.90 | 0.00 | 0.00 |
| 2 | 8.392 | 6728-26-3 | 2-Hexenal,(E)- | C_6_H_10_O | 0.00 | 0.00 | 0.00 | 0.00 | 0.00 | 0.00 | 0.00 | 0.00 | 0.00 | 0.00 | 0.00 | 0.00 | 5.33 |
| 3 | 8.535 | 98-00-0 | 2-Furanmethanol | C_5_H_6_O_2_ | 0.00 | 0.00 | 0.00 | 0.00 | 0.00 | 1.19 | 0.00 | 0.00 | 0.00 | 1.38 | 0.00 | 0.00 | 0.00 |
| 4 | 10.525 | 3208-16-0 | Furan,2-ethyl- | C_6_H_8_O | 0.00 | 0.00 | 0.00 | 0.00 | 0.00 | 0.00 | 0.00 | 0.00 | 0.00 | 0.00 | 0.00 | 0.00 | 2.38 |
| 5 | 12.183 | 100-52-7 | Benzaldehyde | C_7_H_6_O | 4.14 | 4.25 | 5.18 | 9.25 | 7.39 | 11.95 | 9.60 | 8.09 | 7.37 | 11.80 | 9.12 | 7.21 | 7.88 |
| 6 | 13.554 | 3777-69-3 | Furan,2-pentyl- | C_9_H_14_O | 0.00 | 0.00 | 0.00 | 0.00 | 0.00 | 0.00 | 0.00 | 0.00 | 0.00 | 0.00 | 0.00 | 0.00 | 5.86 |
| 7 | 13.633 | 589-98-0 | 3-Octanol | C_8_H_18_O | 0.00 | 1.16 | 0.00 | 0.00 | 0.00 | 0.00 | 0.00 | 0.00 | 0.00 | 0.00 | 0.00 | 0.00 | 0.00 |
| 8 | 14.199 | 4313-03-5 | 2,4-Heptadienal | C_7_H_10_O | 0.00 | 0.00 | 0.00 | 0.00 | 0.00 | 1.21 | 0.00 | 0.00 | 0.00 | 0.00 | 0.00 | 0.00 | 0.00 |
| 9 | 14.953 | 100-51-6 | Benzyl alcohol | C_7_H_8_O | 4.32 | 4.50 | 3.65 | 4.56 | 2.30 | 4.15 | 2.36 | 4.36 | 2.77 | 3.26 | 0.00 | 3.99 | 0.00 |
| 10 | 15.27 | 122-78-1 | Benzeneacetaldehyde | C_8_H_8_O | 4.99 | 6.68 | 4.95 | 4.95 | 5.22 | 6.56 | 5.94 | 4.82 | 5.76 | 7.25 | 4.58 | 5.49 | 0.00 |
| 11 | 17.252 | 78-70-6 | Linolool | C_10_H_18_O | 16.47 | 14.24 | 16.25 | 23.53 | 8.40 | 12.91 | 13.58 | 11.19 | 4.31 | 5.43 | 7.94 | 5.90 | 0.00 |
| 12 | 17.464 | 124-19-6 | Nonanal | C_9_H_18_O | 0.00 | 0.00 | 0.00 | 0.00 | 0.00 | 0.00 | 0.00 | 0.00 | 0.00 | 0.00 | 0.00 | 0.00 | 6.95 |
| 13 | 19.179 | 60-12-8 | Phenylethyl alcohol | C_8_H_10_O | 30.36 | 16.27 | 11.83 | 11.50 | 29.73 | 15.31 | 16.31 | 17.52 | 26.79 | 13.33 | 26.31 | 29.43 | 6.09 |
| 14 | 19.588 | 93-89-0 | Benzoic acid,ethyl ester | C_9_H_10_O_2_ | 7.09 | 5.68 | 2.63 | 4.01 | 0.00 | 0.00 | 0.00 | 1.54 | 0.00 | 1.81 | 0.00 | 0.00 | 0.00 |
| 15 | 19.98 | 91-20-3 | Naphthalene | C_10_H_8_ | 0.00 | 0.85 | 0.00 | 0.00 | 0.00 | 2.70 | 3.99 | 2.26 | 0.00 | 0.00 | 0.00 | 0.00 | 4.62 |
| 16 | 20.559 | 16714-85-5 | 10-Heptadecen-8-ynoic acid, methyl ester | C_18_H_30_O_2_ | 0.00 | 0.00 | 0.00 | 0.00 | 0.00 | 0.00 | 1.35 | 1.38 | 0.00 | 0.00 | 0.00 | 0.00 | 0.00 |
| 17 | 20.607 | 33081-34-4 | Lilac alcohol A | C_10_H_18_O_2_ | 0.00 | 0.00 | 0.00 | 0.00 | 0.00 | 0.00 | 0.00 | 0.00 | 0.00 | 0.00 | 3.14 | 2.61 | 0.00 |
| 18 | 21.003 | 5779-94-2 | Benzaldehyde,2,5-dimethyl- | C_9_H_10_O | 0.00 | 1.44 | 1.22 | 1.87 | 0.00 | 2.46 | 0.00 | 1.93 | 0.00 | 1.74 | 1.24 | 0.00 | 0.00 |
| 19 | 21.195 | 432-25-7 | 1-Cyclohexene-1-carboxaldehyde,2,6,6-trimethyl- | C_10_H_16_O | 0.00 | 1.53 | 1.40 | 0.00 | 0.00 | 1.81 | 0.00 | 1.54 | 0.00 | 0.00 | 1.29 | 0.00 | 4.46 |
| 20 | 22.262 | 103-45-7 | Acetic acid,2-phenylethyl ester | C_10_H_12_O_2_ | 4.91 | 10.25 | 8.55 | 12.07 | 7.72 | 4.83 | 9.43 | 11.64 | 10.62 | 6.92 | 11.08 | 16.22 | 3.05 |
| 21 | 22.923 | 2785-89-9 | Phenol,4-ethyl-2-methoxy- | C_9_H_12_O_2_ | 1.73 | 2.49 | 5.41 | 2.24 | 31.89 | 2.71 | 3.86 | 0.00 | 26.27 | 3.85 | 3.19 | 8.06 | 0.00 |
| 22 | 23.376 | 2443-46-1 | Bicyclo[4.4.1]undeca-1,3,5,7,9-pentaene | C_11_H_10_ | 0.00 | 0.00 | 0.00 | 0.00 | 0.00 | 1.25 | 1.68 | 1.55 | 0.00 | 0.00 | 0.00 | 0.00 | 0.00 |
| 23 | 24.077 | 6155-58-4 | 1,3-Cyclohexadiene-1-Carboxaldehyde,2,6,6-Trimethyl- | C_10_H_14_O | 0.00 | 0.00 | 1.25 | 0.00 | 0.00 | 0.00 | 0.00 | 0.00 | 0.00 | 1.66 | 0.00 | 0.00 | 0.00 |
| 24 | 24.952 | 2021-28-5 | Benzenepropanoic acid,ethyl ester | C_11_H_14_O_2_ | 2.58 | 1.97 | 0.00 | 0.00 | 0.00 | 0.00 | 0.00 | 0.00 | 0.00 | 0.00 | 0.00 | 0.00 | 0.00 |
| 25 | 26.114 | 88395-46-4 | Isospathulenol | C_15_H_24_O | 0.00 | 2.79 | 0.00 | 0.00 | 0.00 | 2.00 | 0.00 | 0.00 | 0.00 | 3.44 | 0.00 | 0.00 | 0.00 |
| 26 | 26.332 | 629-59-4 | Tetradecane | C_14_H_30_ | 0.00 | 0.00 | 1.10 | 0.00 | 0.00 | 0.00 | 0.00 | 0.00 | 0.00 | 0.00 | 0.00 | 0.00 | 0.00 |
| 27 | 26.506 | 1604-34-8 | 2-Undecanone,6,10-dimethyl- | C_13_H_26_O | 0.00 | 0.00 | 0.00 | 0.00 | 0.00 | 0.00 | 0.00 | 0.00 | 0.00 | 1.05 | 0.00 | 0.00 | 0.00 |
| 28 | 26.511 | 575-37-1 | Naphthalene,1,7-dimethyl- | C_12_H_12_ | 0.00 | 1.09 | 0.00 | 0.00 | 0.00 | 0.00 | 0.00 | 0.00 | 0.00 | 0.00 | 0.00 | 0.00 | 0.00 |
| 29 | 26.676 | 14905-56-7 | Tetradecane,2,6,10-trimethyl- | C_17_H_36_ | 0.00 | 0.00 | 0.00 | 0.00 | 0.00 | 4.26 | 6.73 | 3.20 | 0.00 | 7.05 | 4.36 | 0.00 | 0.00 |
| 30 | 27.542 | 77981-89-6 | 2,6,10,10-Tetramethyl-1-oxaspiro[4.5]decan-6-ol | C_13_H_24_O_2_ | 0.00 | 0.00 | 0.00 | 0.00 | 0.00 | 0.00 | 0.00 | 0.00 | 0.00 | 0.72 | 0.00 | 0.00 | 0.00 |
| 31 | 27.808 | 3796-70-1 | 5,9-Undecadien-2-one,6,10-dimethyl-,（E）- | C_13_H_22_O | 0.00 | 0.00 | 0.00 | 0.00 | 0.00 | 2.96 | 2.98 | 5.42 | 0.00 | 4.76 | 0.00 | 0.00 | 0.00 |
| 32 | 28.004 | 3891-99-4 | 2,6,10-Trimethyltridecane | C_16_H_34_ | 0.00 | 2.14 | 1.28 | 0.00 | 0.00 | 2.46 | 2.37 | 2.38 | 0.00 | 0.00 | 2.58 | 2.93 | 0.00 |
| 33 | 28.661 | 97371-50-1 | 4,6,10,10-Tetramethyl-5-oxatricyclo[4.4.0.0(1,4)]dec-2-en-7-ol | C_13_H_20_O_2_ | 0.00 | 1.57 | 1.44 | 1.57 | 0.00 | 1.95 | 5.91 | 0.00 | 2.23 | 2.33 | 2.55 | 0.00 | 0.00 |
| 34 | 28.744 | 79-77-6 | trans-β-Ionone | C_13_H_20_O | 10.68 | 6.95 | 9.27 | 9.63 | 7.37 | 8.20 | 8.32 | 11.72 | 9.52 | 9.95 | 11.04 | 11.27 | 22.32 |
| 35 | 29.723 | 23676-09-7 | Benzoic acid,4-ethoxy-,ethyl ester | C_11_H_14_O_3_ | 0.00 | 0.00 | 3.87 | 0.00 | 0.00 | 0.00 | 0.00 | 0.00 | 0.00 | 0.00 | 0.00 | 0.00 | 7.81 |
| 36 | 29.958 | 17092-92-1 | Dihydroactinidiolide | C_11_H_16_O_2_ | 0.00 | 0.00 | 0.00 | 0.00 | 0.00 | 2.44 | 1.43 | 0.00 | 0.00 | 4.40 | 1.75 | 1.85 | 0.00 |
| 37 | 30.072 | 593-49-7 | Heptacosane | C_27_H_56_ | 0.00 | 0.00 | 0.00 | 0.00 | 0.00 | 0.00 | 0.00 | 1.29 | 0.00 | 0.00 | 0.00 | 0.00 | 0.00 |
| 38 | 31.377 | 106-33-2 | Dodecanoic acid,ethyl ester | C_14_H_28_O_2_ | 1.54 | 0.00 | 1.16 | 0.00 | 0.00 | 0.00 | 0.00 | 0.00 | 0.00 | 0.00 | 0.00 | 0.00 | 0.00 |
| 39 | 34.273 | 2765-11-9 | Pentadecanal- | C_15_H_30_O | 0.00 | 0.00 | 0.00 | 0.00 | 0.00 | 0.00 | 0.00 | 0.00 | 0.00 | 0.00 | 0.00 | 0.00 | 23.26 |
| 40 | 36.031 | 124-06-1 | Tetradecanoic acid,ethyl ester | C_16_H_32_O_2_ | 2.68 | 2.12 | 3.83 | 2.96 | 0.00 | 0.00 | 1.84 | 1.33 | 0.00 | 0.00 | 0.00 | 0.00 | 0.00 |
| 41 | 37.186 | 502-69-2 | 2-Pentadecanone,6,10,14-trimethyl- | C_18_H_36_O | 0.00 | 0.00 | 0.00 | 0.00 | 0.00 | 0.00 | 0.00 | 0.00 | 0.00 | 1.72 | 0.00 | 0.00 | 0.00 |
| 42 | 37.591 | 102608-53-7 | 3,7,11,15-Tetramethyl-2-hexadecen-1-ol | C_20_H_40_O | 0.00 | 2.66 | 0.00 | 0.00 | 0.00 | 0.00 | 0.00 | 0.00 | 0.00 | 0.00 | 0.00 | 0.00 | 0.00 |
| 43 | 38.505 | 2922-51-2 | 2-Heptadecanone | C_17_H_34_O | 0.00 | 0.00 | 5.15 | 5.04 | 0.00 | 1.77 | 0.00 | 3.21 | 0.00 | 3.72 | 4.24 | 3.28 | 0.00 |
| 44 | 38.518 | 14852-31-4 | 2-Hexadecanol | C_16_H_34_O | 0.00 | 0.00 | 0.00 | 0.00 | 0.00 | 0.00 | 0.00 | 0.00 | 0.00 | 0.88 | 0.00 | 0.00 | 0.00 |
| 45 | 41.074 | 628-97-7 | Hexadecanoic acid,ethyl ester | C_18_H_36_O_2_ | 8.49 | 9.38 | 10.58 | 6.83 | 0.00 | 1.96 | 0.00 | 3.62 | 0.00 | 0.00 | 2.68 | 1.76 | 0.00 |
