# Supplementary material for: Effects of Lactic Acid Bacteria Additives on the Quality, Volatile Chemicals and Microbial Community of Leymus chinensis Silage During Aerobic Exposure
Source: Front Microbiol. 2022 Sep 2;13:938153. doi: 10.3389/fmicb.2022.938153 (PMC9478463; doi:10.3389/fmicb.2022.938153)
Supplement: Supplementary file 2 [file Table_2.DOC]

S 2 VIP scores and P values for differentially volatile chemicals

| LP-CK  R2X(cum):0.664  R2Y(cum):0.993  Q2(cum):0.981 | | | LB-CK  R2X(cum):0.616  R2Y(cum):0.987  Q2(cum):0.951 | | | PB-CK  R2X(cum):0.734  R2Y(cum):0.988  Q2(cum):0.957 | | |
| --- | --- | --- | --- | --- | --- | --- | --- | --- |
| Var ID | VIP Score | P value | Var ID | VIP Score | P value | Var ID | VIP Score | P value |
| C_15_H_24_O | 1.55786 | 0.000 | C_16_H_34_ | 1.81497 | 0.000 | C_17_H_34_O | 1.77267 | 0.000 |
| C_8_H_10_O | 1.3963 | 0.000 | C_11_H_16_O_2_ | 1.45298 | 0.002 | C_10_H_12_O_2_ | 1.3859 | 0.000 |
| C_9_H_10_O | 1.32431 | 0.000 | C_17_H_36_ | 1.43001 | 0.004 | C_16_H_34_ | 1.35318 | 0.001 |
| C_5_H_6_O_2_ | 1.22997 | 0.002 | C_10_H_16_O | 1.4105 | 0.001 | C_9_H_10_O | 1.27697 | 0.025 |
| C_16_H_34_ | 1.22605 | 0.001 | C_9_H_10_O | 1.36266 | 0.001 | C_9_H_12_O_2_ | 1.24869 | 0.005 |
| C_10_H_16_O | 1.22422 | 0.001 | C_17_H_34_O | 1.35473 | 0.003 | C_10_H_16_O | 1.24802 | 0.064 |
| C_17_H_34_O | 1.20942 | 0.004 | C_8_H_10_O | 1.30673 | 0.010 | C_10_H_8_ | 1.24603 | 0.064 |
| C_17_H_36_ | 1.18296 | 0.005 | C_9_H_12_O_2_ | 1.30424 | 0.005 | C_11_H_10_ | 1.24276 | 0.066 |
| C_9_H_12_O_2_ | 1.17205 | 0.003 | C_13_H_20_O_2_ | 1.29077 | 0.049 | C_13_H_22_O | 1.22987 | 0.069 |
| C_13_H_20_O_2_ | 1.06894 | 0.009 | C_10_H_14_O | 1.12954 | 0.071 | C_18_H_30_O_2_ | 1.22479 | 0.070 |
| C_12_H_12_ | 1.04319 | 0.064 | C_14_H_30_ | 1.0832 | 0.064 | C_27_H_56_ | 1.17925 | 0.081 |
| C_8_H_18_O | 1.02915 | 0.067 | C_11_H_14_O_3_ | 1.03547 | 0.083 |  |  |  |
| C_20_H_40_O | 1.02306 | 0.071 |  |  |  |  |  |  |
| C_10_H_8_ | 1.01902 | 0.010 |  |  |  |  |  |  |
|  |  |  |  |  |  |  |  |  |
